# Gut Microbiota and Nonalcoholic Fatty Liver Disease: Insights on Mechanisms and Therapy

**DOI:** 10.3390/nu9101124

**Published:** 2017-10-16

**Authors:** Junli Ma, Qihang Zhou, Houkai Li

**Affiliations:** Center for Traditional Chinese Medicine and Systems Biology, Institute for Interdisciplinary Medicine Sciences, Shanghai University of Traditional Chinese Medicine, Shanghai 201203, China; flyingwalnuts@163.com (J.M.); 15221368273@163.com (Q.Z.)

**Keywords:** gut microbiota, obesity, insulin resistance, NAFLD, probiotic, prebiotic, symbiotic

## Abstract

The gut microbiota plays critical roles in development of obese-related metabolic diseases such as nonalcoholic fatty liver disease (NAFLD), type 2 diabetes(T2D), and insulin resistance(IR), highlighting the potential of gut microbiota-targeted therapies in these diseases. There are various ways that gut microbiota can be manipulated, including through use of probiotics, prebiotics, synbiotics, antibiotics, and some active components from herbal medicines. In this review, we review the main roles of gut microbiota in mediating the development of NAFLD, and the advances in gut microbiota-targeted therapies for NAFLD in both the experimental and clinical studies, as well as the conclusions on the prospect of gut microbiota-targeted therapies in the future.

## 1. Introduction

The mammalian gastrointestinal tract is the main site for commensal bacteria. There are over 10^14^ microorganisms inside human body [1], which play important roles in maintaining human health [2]. The abundance and composition of gut microbiota are highly variable in the context of different conditions contributing to the development of various diseases [3,4]. In recent years, a huge number of studies have revealed the critical roles of gut microbiota in the development of metabolic diseases including type 1 and 2 diabetes [5,6], obesity [7,8,9,10], cardiovascular disease [11,12,13], and chronic liver diseases [14]. 

Nonalcoholic fatty liver disease (NAFLD) is a spectrum of chronic liver diseases including simple steatosis, nonalcoholic steatohepatitis (NASH), fibrosis, cirrhosis, and hepatocellular carcinoma (HCC) [9]. NAFLD is the most common chronic liver disease due to the prevalence of obesity worldwide [15]. In addition to the well-established “two-hit” theory [16], the alteration of gut microbiota also promotes the development of NAFLD by mediating processes of inflammation, insulin resistance, bile acids, and choline metabolism [17,18]. As a result, the elucidation on the roles of gut microbiota in NAFLD highlights the significance of gut microbiota-targeted therapies for NAFLD [19,20]. There are various ways to manipulate gut microbiota, for example through the use of probiotics, prebiotics, synbiotics, antibiotics, and some active components from herbal medicines. 

In this review, we retrieved the publications on the topics of gut microbiota and NAFLD mainly published within the past 10 years through Pubmed. Based on all of the publications available, we first reviewed the main roles of gut microbiota in mediating NAFLD formation. Then, we discussed the status of gut microbiota-targeted therapies in NAFLD with both the experimental and clinical evidence, and made conclusions on the therapeutic potential of manipulating gut microbiota in the future.

## 2. Roles of the Gut Microbiota in NAFLD Development

Obesity is the common ground of most metabolic diseases. The gut microbiota plays critical roles in the development of obesity and obese-related metabolic diseases [21] by producing microbial metabolites like short-chain fatty acids (SCFAs) that regulate host energy harvest [22,23], or by modulating signaling pathways of host energy metabolism [24]. Study has revealed that the gut microbiota promotes the intestinal absorption of monosaccharides, accelerating de novo hepatic lipogenesis and suppressing fasting-induced adipocyte factor, resulting in the accumulation of triglycerides in adipocytes [25]. More evidence of gut microbiota affecting host energy metabolism has been acquired in numerous studies [25,26,27]. 

Insulin resistance is a basic pathophysiological process of metabolic diseases [28,29]. In NAFLD, insulin resistance accelerates the fat accumulation and inflammation in hepatocytes [30]. The enhanced inflammation and insulin resistance forms a “vicious cycle” deteriorating the development of NAFLD. The gut epithelium is a natural barrier for preventing the translocation of detrimental bacteria and harmful elements into circulation. NASH patients are typically characterized with small intestine bacterial overgrowth (SIBO) that may impair the intestinal tight junction and subsequently increase intestinal permeability. SIBO also induces hepatic expression of toll like receptor 4 (TLR4) and release of interleukin (IL)-8 that stimulates inflammatory reaction. The term “metabolic endotoxemia” was coined because of increased lipopolysaccharide (LPS) levels in the circulation in metabolic diseases [31], in which LPS combines with LPS binding protein (LBP) and then binds to themonocyte differentiation antigen(CD14)-TLR-4complex triggering an inflammatory reaction and insulin resistance [32,33,34]. Therefore, gut dysbiosis is causative for enhanced secretion of LPS and its mediated inflammation in NAFLD development. 

Choline not only is an indispensable component of cell membrane phospholipids, but also plays important role in lipid metabolism. Choline facilitates the lipid transport in hepatocytes and prevents the abnormal accumulation of lipids in the liver, while choline deficiency usually leads to hepatic steatosis [35,36]. The gut microbiota is also involved in choline metabolism by converting it into toxic dimethylamine and trimethylamine, which are transported to liver and converted into trimethylamine oxide (TMAO) that causes liver inflammation and damage [37]. The increased production of TMAO is also the culprit for cardiovascular disease [37,38,39]. On the other hand, the content of dietary choline influences the composition and abundance of gut microbiota that are associated with the development of NAFLD [40]. The close relationship between gut microbiota and choline metabolism provides an important rationale for gut microbiota-targeted therapy for NAFLD.

**Figure 1:**
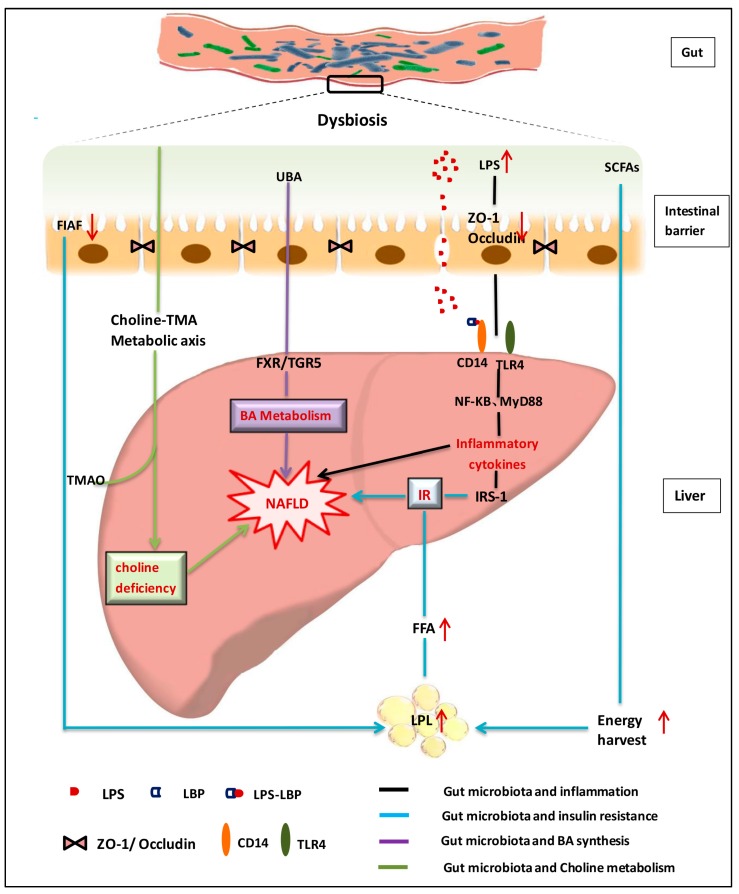
Schematic view on roles of gut microbiota in nonalcoholic fatty liver disease [2,48,49,50,51,52,53,54,55,56,57,58,59,60]. NAFLD: nonalcoholic fatty liver disease; LPS: lipopolysaccharides; LBP: LPS binding protein; SCFAs: short chain fatty acids; BA:Bile acid; TNF-α: tumor-necrosis factor alpha; TLR: Toll like receptor; CD14: monocyte differentiation antigen; UBA: unconjugated bile acid; ZO-1\Occludin: two tight junction proteins; FIAF: Fasting-induced adipocyte factor; NF-kB: Nuclear factor-κB; MyD88: myeloid differentiation factor 88; FXR: farnesoid X receptor; TGR5: Takeda G protein-coupled receptor 5; TMA: trimethylamine; TMAO: trimethylamine oxide; IR: Insulin resistance; IRS: Insulin receptor substrate; FFA: free fatty acid; LPL: lipoprteinlipase.

Bile acids are synthesized from cholesterol with a wide range of physiological functions. Bile acids can not only facilitate digestion and absorption of fat-soluble food, but also preserve the intestinal barrier and prevent bacterial translocation [41,42]. Moreover, bile acids could function as signaling molecules that modulate the balance of bile acids metabolism by activating the farnesoid X receptor (FXR) and G protein-coupled bile acid receptor(TGR5) [43,44,45,46]. Studies reveal that antibiotics could attenuate the high-fat diet-induced NAFLD development by altering the composition of bile acids and inhibiting the FXR signaling pathway, whereas mice with intestine-specific FXR disruption have reduced triglyceride accumulation in the liver compared with control mice [47]. Bile acids usually have strong anti-microbial properties and gut microbiota can influence the homeostasis of bile acids pool by deconjugating and metabolizing the primary bile acids into secondary bile acids in the intestinal tract, which are involved in modulating lipids and energy metabolism pathways during NAFLD formation [44]. The crosstalk between gut microbiota and bile acids provides fundamental evidence for gut microbiota-targeted therapy of NAFLD. A schematic view on the roles of gut microbiota on NAFLD formation is summarized in Figure 1. 

## 3. Gut Microbiota-Targeted Therapies in NAFLD

NAFLD is common with the current prevalence of obesity, however, clinical therapeutic options are still very scarce with respect to safety, effectiveness, and patient compliance [61]. As a result, the intricate relationship between gut microbiota and NAFLD opens up a new window for seeking effective and safe therapies on NAFLD by restoring gut homeostasis of NAFLD patients in various ways. 

### 3.1. Gut Microbiota-Targeted Therapy with Probiotics

Probiotics are a collection of bacteria with a wide range of beneficial effects on host metabolism [2,62]. Bacteria of *Lactobacillus*, *Bifidobacterium* and *Satreptococcus* are most frequently used probiotics that can inhibit expansion of gram-negative pathogenic bacteria [63]. Okubo et al. investigated the effects of *Lactobacillus caseistrain* Shirota (LcS) on methionine-choline-deficient (MCD) diet-induced NASH mice [64]. They found that the MCD diet resulted in significant reduction in lactic acid bacteria (*Bifidobacterium* and *Lactobacillusin*) in feces, but values were increased by LcS supplementation. Moreover, the LcS supplement dramatically improved the symptoms of NASH induced by MCD such as hepatic histology and serum parameters triglycerides (TG), total cholesterol (TC), as well as the altered expression of hepatic genes and proteins (the mRNA levels of actin alpha/alpha-SMA(α-SMA) and tissue inhibitor of metalloproteinase 1(TIMP-1)). Meanwhile, metabolic beneficial effects of LcS supplement were observed in high-fat diet (HFD)-induced and genetic *db/db* obese mice, in which LcS supplementation significantly improved insulin resistance and lowered plasma levels of LBP [65]. Study revealed that LcS treatment protected against the fructose-induced NAFLD by suppressing the activation of the TLR4 signaling cascade in the liver [66]. Accordingly, the beneficial effect of LcS in metabolic diseases is due to the improvement of metabolic endotoxemia.

*Lactobacillus* is a genus of gram-positive bacteria which can convert sugars into lactic acid. Bacteria from *Lactobacillus* genus have been trialed as probiotics in studies [67,68,69]. Sohn et al. investigated the effects of *Lactobacillus paracasei* on NASH patients [70] and found that *L. paracasei* administration lowered inflammatory cytokines in NASH patients. However, probiotics with a single species of *Lactobacillus* bacteria did not show benefit in patients with irritable bowel syndrome or Crohn’s disease [71,72]. Meanwhile, the beneficial effects of *Lactobacillus plantarum* probiotics were investigated in NAFLD models such as *L. plantarum* MA2, *L. plantarum* A7 and *L. plantarum* NCU116. Results showed that either *L. plantarum* A7 or *L. plantarum* MA2 was effective in lowering serum lipids [73,74], while *L. plantarum* NCU116 improved liver function and decreased hepatic fat accumulation as well [75]. A similar effect was observed with *L. rhamnosus* supplementation in an NAFLD model. Probiotics of *L. rhamnosus* GG (LGG) protected mice from NAFLD by increasing the abundance of beneficial bacteria, improving gut barrier function and attenuating hepatic inflammation [76], as well as the cholesterol-lowering effect through inhibition of the FXR and FGF15(fibroblast growth factor) signaling pathway [77]. In addition, several other species of *Lactobacilli* bacteria have shown potential in NAFLD prevention, including *L. johnsonii* BS15 [78], *L. reuteri* GMNL-263 [79], *L. gasseri* BNR17 [80]. 

*Bifidobacterium* (*Bif*) belongs to the *Bifidobacteria* bacteria genera in the mammalian gastrointestinal tract, and is a frequently used probiotic [81,82,83]. Supplementation of *Bif* significantly improved visceral fat accumulation and insulin sensitivity in HFD-fed rats [84]. Administration of *Bifidobacterium pseudocatenulatum* CECT 7765 could reduce serum cholesterol and triglycerides, and improved glucose tolerance in obese mice [85]. It is proposed that probiotic of *Bif* is superior to *Lactobacillus acidophilus* in reducing hepatic fat accumulation [86]. Compared to probiotics with a single strain of bacteria, VSL#3 is a mixed probiotic with eight types of bacteria (*Bifidobacteria* (*B. breve*, *B. longum*, *B. infantis*), *Streptococcus thermophilus*, *L. plantarum*, *L. acidophilus*, *L. paracasei* and *L. delbrueckii* subsp. *bulgaricus*) which has shown great potential in treatment of various diseases [87,88,89,90,91]. Experimental evidence has indicated that VSL#3 could attenuate inflammation via modulation of the nuclear factor-kB (NF-kB) pathway [92], reduce hepatic fat accumulation and ALT levels [93], improve insulin sensitivity in NAFLD models [94], and prevent against liver fibrosis in NASH patients [95]. The probiotic with combined bacteria (LGG, *Lactobacillus plantarum* WCFS1 and anthraquinone from *Cassia obtusifolia* L.) was effective in reducing blood lipid levels and improving insulin resistance in NAFLD rats [96]. Meanwhile, supplementation of combined probiotic (*Bifidobacterium infantis*, *Lactobacillus acidopilus,* and *Bacillus cereus*) could improve gut dysbiosis and liver function via suppression of the LPS/TLR4 signaling pathway [97]. Kim et al. found that consumption of kefir (a probiotic beverage containing over 50 species of lactic acid bacteria and yeast) prevented obesity and NAFLD formation by restoring the gut microbiota and enhancing fatty acid oxidation in HFD-fed mice [98]. Further evidence of beneficial effects on NAFLD prevention has been acquired in many studies by administering probiotics with mixed bacteria [99,100,101]. In addition to the direct impacts on the composition of gut microbiota, the beneficial effects of probiotics on NAFLD are also associated with their metabolic activities [53]. It has been reported that probiotics of clostridium butyricum MIYAIRI 588—a butyrate-producing bacteria, decreased accumulation of lipid droplets in HFD-induced NAFLD models, improved insulin resistance [102], and reduced hepatic lipids and serum endotoxin levels in choline-deficient/l-amino acid-defined diet-induced NAFLD models [103], which may be associated with the stimulation of expression of adenosine 5′-monophosphate (AMP)-activated protein kinase (AMPK) andserine/threonine kinas (AKT) proteins, and lipogenesis- or lipolysis-related proteins.

Currently, although the beneficial effects of probiotics were mainly acquired in experimental studies, some consistent results have also been observed in clinical practice. Alisi *et al*. compared the therapeutic effects of VSL#3 in a randomized double-blind controlled study in obese children with biopsy-proven NAFLD [104]. They found that 4-month supplement of VSL#3 significantly improved liver function and increased glucagon-like peptide (GLP-1)/active glucagon-like peptide (aGLP1) levels suggesting the effects of VSL#3 might be GLP-1-dependent. Consistent effects were also observed on obese children with NAFLD by administering probiotics such as *Lactobacillus rhamnosus* strain GG [105] and mixed bacteria of *Lactobacillus bulgaricus* and *Streptococcus thermophilus* [106]. Sepideh et al. investigated the effects of a multistrain probiotic supplementation in NAFLD patients in a RCT study, and the results showed dramatic improvement in insulin sensitivity and inflammation [107]. Moreover, synergistic effects were also observed by combining probiotics with chemical drugs such as metformin in NASH and statins in NAFLD therapy [108,109], which highlights the great potential of clinical application of probiotics either alone or combined with other drugs. Nevertheless, the clinical efficacy of probiotics still needs further validation in well-designed studies with a larger scale of participants. Solga et al. observed that 4 months of probiotic supplements not only did not reduce hepatic steatosis, but increased fat accumulation in liver of four patients [110]. In 2010, Andreasen et al. conducted a randomized-double-blinded research on effects of *L. acidophilus* NCFM on insulin sensitivity and the systemic inflammation [111]. They found that insulin sensitivity was improved in the probiotic group, but not in the placebo group, and there were no differences in systemic inflammation in either group. Meanwhile, another study indicated that an 8-week probiotic supplement did not improve total cholesterol, low-density lipoprotein (LDL)-cholesterol, high-density lipoprotein (HDL)-cholesterol, TG, TG/ LDL and LDL/HDL ratios in diabetic patients [112]. Additionally, supplementation with *Lactobacillus acidophilus* did not improve the levels of plasma lipids in volunteers with elevated cholesterols in a double-blind placebo-controlled study [113]. A detailed summary of gut microbiota-targeted therapies on NAFLD with probiotics is provided in Table 1.

### 3.2. Gut Microbiota-Targeted Therapy with Prebiotic

Prebiotics are indigestible food ingredients with beneficial effects, as they selectively stimulate the growth and/or activity of “good” and suppress the “bad” bacteria resident in the colon [123]. They can be defined as a fermented ingredient that allows changes both in the composition and/or activity in the gastrointestinal microflora conferring benefits upon host well-being and health [124,125]. Evidence suggested that prebiotic supplements prevented NAFLD development in both experimental and clinical studies [126,127]. 

In 2009, Cani et al. found that prebiotics of oligofructose (a mixture of fermentable dietary fibers) decreased plasma LPS and cytokine levels, and hepatic expression of inflammatory and oxidative stress markers in obese mice. An improvement in intestinal permeability and production of GLP-2 was also shown [128]. In an MCD diet-induced steatohepatitis mice model, a dietary fructooligosaccharide (FOS) supplement attenuated the extent of steatohepatitis by restoring the homeostasis of gut microbiota and intestinal epithelial barrier function [129]. Pachikian et al. reported that a FOS supplement reduced hepatic triglyceride accumulation in *n*-3 PUFA (polyunsaturated fatty acid)-depleted diet-induced NAFLD model by altering microbiota composition and increasing production of GLP-1 [130]. Meanwhile, the FOS supplement stimulated fatty acid oxidation by activating peroxisome proliferator-activated receptor-alpha (PPAR-α) and reduced cholesterol accumulation by inhibiting SREBP-2 (sterol-regulatory-element-binding protein isoform 2) in liver without affecting SREBP-1 expression and activity [130,131]. Lactulose is a prebiotic that promotes the growth of lactic acid bacteria and *Bifidobacteria* [132]. A study indicated that lactulose treatment decreased the hepatic inflammation and serum endotoxin levels in rats with steatohepatitis [133]. Chitin–glucan (CG) is another type of prebiotic from fungal source. Neyrinck et al. investigated the function of CG in HFD-induced obese mice and found CG treatment decreased body weight gain, improved glucose intolerance and hepatic triglyceride accumulation by restoring bacteria of clostridial cluster XIVa [134]. 

The combination of prebiotics with natural components will yield more benefits than prebiotics on their own. For example, combined therapy of isomalto-oligosaccharides (IMOs) with lycopene (an antioxidant) prevented body weight gain, enhanced adipose tissue fat mobilization, and improved insulin resistance and metabolic endotoxemia in HFD-induced NAFLD mice. The observed effects were associated with their modulation of microbial production of SCFAs [135].

In the clinic, prebiotics have also been tested for their benefits in various diseases [136,137,138,139,140]. Oligofructose (OFS), an inulin-type fructan, was added to diet of NASH patients in a pilot randomized double-blind study [127]. Their results showed that the OFS supplement decreased serum ALT and AST levels significantly. Prebiotics of mixed galacto-oligosaccharides and fructo-oligosaccharides (9:1) stimulated the abundance of *Bifidobacteria* bacteria in infants [141]. Similarly, administration of prebiotic inulin and oligofructose (50:50 in mixture) increased the abundance of *Bifidobacterium* and *Faecalibacterium prausnitzii*, which negatively correlated with serum LPS levels [142]. Prebiotics have shown great potential in prevention of obesity and NAFLD development by lowering the permeability of intestinal wall, attenuating metabolic endotoxemia, and reducing the accumulation of fat [143]. The gut microbiota-targeted therapies with prebiotics were summarized in Table 2.

### 3.3. Gut Microbiota-Targeted Therapy with Synbiotic

Synbiotics are the combination of probiotics and prebiotics [144]. Synbiotics usually produce benefits by selectively stimulating the growth and/or activating the metabolism of health-promoting bacteria [145]. Administration of synbiotics containing *Lactobacillus paracasei* B21060 plus arabinogalactan and fructooligosaccharides attenuated hepatic inflammation and increased expression of nuclear PPARs and their targeted genes in HFD-induced NAFLD rats [146]. Synbiotics have shown various benefits in metabolic diseases, such as improvement of insulin resistance, glucose control, and inflammatory cytokine synthesis [147,148,149]. 

In the clinic, the therapeutic effect of a synbiotic containing seven probiotics and oligofructose was evaluated in patients with NAFLD in a double-blind RCT. The results showed that synbiotic therapy significantly decreased ALT levels [150]. Malaguarnera et al. observed that combination of synbiotic (*B. longum* and Fos) and lifestyle intervention in NASH patients resulted in a much greater improvement compared to lifestyle intervention alone, including reduction of serum TNFα, CRP (C-reactive protein), endotoxin, and AST levels, improvement in HOMA-IR( homeostasis model assessment of insulin resistance)and extent of NASH activity index [151]. Synbiotic therapy showed improvements in levels of fasting blood glucose, TG, and inflammatory cytokines in both obese and lean NAFLD patients [152,153]. Therefore, synbiotics are a promising gut microbiota-targeted intervention for NAFLD prevention or therapy. Nevertheless, more clinical validations are also needed. A summarized gut microbiota-targeted therapy on NAFLD with synbiotics was provided in Table 3. 

### 3.4. Gut Microbiota-Targeted Therapies with Other Approaches

In addition to probiotics/prebiotics/synbiotics, gut microbiota-targeted interventions have also been investigated with other approaches. Butyrate is an SCFA and is an important gut microbial metabolite derived from fermentation of nondigestible polysaccharides. Butyrate has a critical role in affecting metabolic disease development in a variety of ways, including modulation on energy harvest, hepatic lipogenesis and gluconeogenesis, adipokine signaling in adipocytes, intestinal permeability, and appetite regulation in the brain [154,155]. Administration of sodium butyrate alleviated inflammation and fat accumulation in HFD-induced NAFLD mice by increasing the abundances of the beneficial bacteria *Christensenellaceae*, *Blautia* and *Lactobacillus* [156]. Therefore, appropriate approaches such as engineered bacteria could be developed to enhance the production of beneficial gut microbial metabolites (e.g., butyrate) or intervention with chemical drugs to promote the proliferation of “good” bacteria, and suppress the “bad” ones. 

Antibiotics are frequently used in the clinic, although their disruption of gut microbial homeostasis is a double-edged sword [157]. On one hand, the short-term application of antibiotic can result in long-lasting impacts on host metabolism. On the other hand, administration of some kinds of antibiotics may attenuate diseases. For example, oral administration of cidomycin increased the small intestine transit rate and lowered serum ALT, AST, and TNF-α levels in NASH rats, suggesting the potential of cidomycin in alleviating the severity of NASH by intervening gut microbiota [158]. In the clinic, administration of rifaximin could decrease the circulating endotoxin and ALT levels in patients with NAFLD [159]. Although the improvement in NAFLD, especially in NASH, by short-term administration of antibiotic (e.g., rifaximin) can be observed, the long-term application of antibiotics is not encouraged because of probable side effects [160]. Nevertheless, the changes in gut microbiota resulting from antibiotics could provide important evidence for exploring alternative ways to modulate gut microbiota in disease therapy.

Compared to antibiotics, some ingredients from herbal medicines have shown more prospects for gut microbiota modulation with minor side effects [161,162]. Berberine is a typical herbal component with potent antibacterial activity, especially bacteria in intestinal tract, because berberine can hardly be absorbed in gut [163]. Currently, increasing evidence has confirmed the therapeutic effect of berberine on metabolic diseases including obesity, NAFLD, and type 2 diabetes via modulation on gut microbiota [164,165,166]. It has been revealed that berberine administration restored the relative abundance of *Bifidobacteria* and the ratio of *Bacteroidetes*/*Firmicutes* in HFD-induced NASH mice resulting in significant reduction in body weight, serum levels of lipids, glucose, insulin and inflammatory cytokines [167,168]. TSG (2,3,5,4′-tetrahydroxy-stilbene-2-*O*-β-d-glucoside) is an active component of the traditional Chinese medicine (TCM) *Polygonum multiflorum* Thunb, which has shown significant effects in NAFLD prevention by modulating gut microbiota, improving the intestinal mucosal barrier, and suppressing the expression of NF-κB [169]. Resveratrol is a natural polyphenol with anti-oxidative activity [170]. Recent studies showed resveratrol was also effective in preventing metabolic diseases such as obesity and NASH by regulating gut microbiota [171]. In addition to the individual component from herbal medicines, recent investigations revealed that the efficacy of some TCM formulas was associated with the modulation on gut microbiota. For example, Qushi Huayu Fang (a mixture of five herbs including *Artemisia capillaries Thunb*, *Gardenia jasminoides Ellis*, *Fallopia japonica*, *Curcuma longa* L., and *Hypericum japonicum Thunb*) is an ancient TCM formula which has been used for NAFLD treatment. Recent studies showed that administration of Qushi Huayu Decoction (QHD) significantly decreased body weight, alleviated hepatic steatosis, and reduced the content of TG and free fatty acids in liver in HFD-induced NAFLD rats. It showed that the QHD-treated group harbored significantly different gut microbiota from that of model rats, and the bacterial profiles of NAFLD rats could be modulated by the QHD [172,173]. Recently, the anti-obesity property of daesiho-tang (DSHT) was also investigated. It was found that DSHT treatment significantly reduced levels of serum TC and TG as well as hepatic fat accumulation that were associated with the regulation on abundance of gut microbiota [174]. Although the mechanisms underlying TCM therapy are extremely complicated and largely unknown, the gut microbiota was supposed to be an important target for many TCM formulas because many kinds of chemicals derived from TCM are unabsorbable. Those unabsorbed chemicals in TCM can influence gut microbiota directly or be metabolized into absorbable or active form by gut microbiota. A summary of gut microbiota-targeted therapies on NAFLD with other approaches were provided in Table 4. 

## 4. Conclusions and Perspectives

Currently, the gut microbiota has been recognized as a critical factor contributing to the development of NAFLD and the gut microbial-related mechanisms have also been well elucidated. As a result, the strategy of gut microbiota-targeted therapy on NAFLD is highly valued in the context of accumulating benefits of gut microbial modulation by using probiotics, prebiotics, synbiotics, antibiotics, and herbal medicines. Although many experimental reports were exciting, discrepant results were also observed in the clinic. Therefore, the clinical efficacy of gut microbiota-targeted therapies on NAFLD still need to be confirmed with large-scale and well-organized RCT studies. The main factors contributing to the variation of therapeutic outcomes in the clinic are differences in bacterial activity of probiotics or the diversified dysbiosis among NAFLD patients. In this sense, probiotics with mixed bacteria such as VSL#3 are more prospective than those with individual type of bacteria. Meanwhile, the gut microbiota-related efficacy of natural components from herbal medicines or the TCM formula itself highlighted the great potential of seeking novel medicines from TCM because some TCMs showed their effects by nourishing “good” bacteria and suppressing “bad” ones. Currently, 16S rDNA-based sequencing is still the major approach for most gut microbiota-involved studies because it is relatively affordable and applicable for most laboratories. Although 16S rDNA sequencing can provide a general description on the structural differences of the microbiome between samples, especially at the genus level, it is usually frustrating when information for specific bacteria species is necessary. Consequently, metagenomics will be more applicable for figuring out specific bacterial species that may contribute to the disease development or therapeutic efficacy, as well as the involved microbial functions. 

In summary, gut microbiota-targeted therapies for diseases are still in their infancy. Nevertheless, we envision that more gut microbiota-targeted therapies will be tested in the context of accumulation of therapeutic evidence and advances in elucidation of gut microbial-related mechanisms in diseases, as well as the technological innovation of gut microbiome analysis. 

## Figures and Tables

**Table 1:** Gut microbiota-targeted therapies of NAFLD with probiotics.

Interventions		Main Effects	Experimental Models	Ref.
**Probiotic**	*Lactobacillus* (LcS)	Suppressing NASH development	MCD diet-induced NASH in mice	[64]
		Improving insulin resistance and glucose intolerance	Diet-induced obesity (DIO) mice.	[65]
		Protecting against the onset of fructose-induced NAFLD	Fructose-induced NAFLD in mice	[66]
	*L*. *paracasei*	Attenuating hepatic steatosis	(High fat +10% fructose diet)-induced NASH in mice	[70]
	*L. plantarum* A7	Lowering serum lipids, TC, and TG levels	High-cholesterol diet-fed rats	[73]
	*L. plantarum* MA2	Lowering serum TC, TG and low-density lipoprotein cholesterol	Cholesterol-enriched diet-fed rats	[74]
	*L. plantarum* NCU116	Improving liver function, oxidative stress and lipid metabolism	HFD-induced NAFLD in rats	[75]
	*Lactobacillus rhamnosus* GG (LGG)	Protecting mice from NAFLD attenuated liver inflammation and steatosis	High-fructose diet induced NAFLD in mice	[76]
		Improving NAFLD	HFD-induced NAFLD in rats	[82]
		Improving in alanine aminotransferase levels	20 obesity-related liver abnormalities in children	[105]
	*L. johnsonii* BS15	Effective in preventing NAFLD	HFD-induced NAFLD in mice	[78]
	*L. reuteri* GMNL-263	Ameliorating hepatic steatosis	High-fructose diet-fed rats	[79]
	*L. gasseri* BNR17	Inhibiting increases in body and adipocyte tissue weight	High-sucrose diet-induced obese mice.	[80]
	3 *Lactobacillus* strains	Reducing serum TC, TG, and low-density lipoprotein cholesterol	HFD-fed rats	[114]
	*L. acidophilus* NCFM	Inflammatory markers and the systemic inflammatory response were unaffected	45 males with T2D	[111]
	*L. acidophilus*	No changes in serum lipids	80 patients with elevated cholesterols	[113]
	*Bifidobacterium* (*Bif*)	Ameliorating visceral fat accumulation and insulin sensitivity	HFD-fed rats	[84]
		Attenuating hepatic fat accumulation	HFD-induced NAFLD in rats	[86]
		Reducing body and fat weights, blood serum levels (TC, HDL-C, LDL-C, TG, AST, ALT, and lipase levels)	HFD-induced obesity in rats	[115]
	*B. pseudocatenulatum* CECT 7765	Reducing serum cholesterol, TG, and insulin resistance	HFD-fed mice	[85]
	*Bacteroides uniformis* CECT 7771	Reducing body weight gain, liver steatosis and cholesterol and TG concentrations	HFD-induced obesity mice	[116]
**Probiotic**	VSL#3	Limiting oxidative and inflammatory liver damage	HFD-fed young rats	[92]
		Reducing hepatic total fatty acid content and ALT levels.	HFD-induced NAFLD in mice	[93]
		Improvements in steatosis and insulin resistance	HFD-fed mice	[94]
		Modulating liver fibrosis, without protecting from inflammation and steatosis in NASH.	MCD diet-induced NASH in mice.	[95]
		Improving the degree of liver disease in children	44 Obese children with NAFLD	[104]
		Improving plasma levels of lipid peroxidation markers: MDA(malondialdehyde), 4-HNE( 4-hydroxynonenal).	22 patients with NAFLD + 20 patients with AC (alcoholic liver cirrhosis )	[117]
		Experiencing a significant increase in liver fat; no significant differences in any of the blood assays or clinical parameters	4 patients with NAFLD	[110]
	Probiotic mixtures	Improving NAFLD	HFD-induced NAFLD in rats	[96]
		Delaying the progression of NAFLD via LPS/TLR4 signaling	HSHF diet-induced NAFLD in rats	[97]
		Improving NAFLD pathogenesis and steatosis	High fat and sucrose diet (HFSD)-induced NAFLD in rats	[118]
		Influencing protein expression and decreasing steatohepatitis	MCD diet-induced NASH in rats	[99]
		Reducing obesity-related biomarkers and modulating the microbial community	Obese mice	[100]
		Modulating gut microbiota and up-regulated genes related to fatty acid oxidation in both the liver and adipose tissue	HFD-induced obese mice	[98]
		Improving liver aminotransferases levels	30 patients with NAFLD	[106]
		Decreasing levels of ALT and AST and improving pediatric NAFLD	64 obese children with NAFLD	[119]
		Reducing insulin, insulin resistance, TNF-a, and IL-6	42 patients with NAFLD	[107]
		No significant changes in (LDL)-cholesterol, (HDL)-cholesterol, TG, TC TG/LDL and LDL/HDL ratios	60 patients with T2DM	[112]
		Great reductions in serum AST level and liver fat	20 patients with NASH	[120]
	MIYAIRI 588	Improving NAFLD and decreasing accumulation of lipid droplets	HFD-induced NAFLD in rats	[102]
		Improving hepatic lipid deposition and decreasing the triglyceride content, insulin resistance, serum endotoxin levels, and hepatic inflammatory indexes.	Choline-deficient/ l-amino acid-defined (CDAA)-diet-induced NAFLD in rats	[103]
	Probiotics and metformin	Improvements in liver aminotransferases, cholesterol, and TG	64 patients with NASH	[108]
	Probiotics and statins	Lowering cholesterol and products of metabolism of intestinal microflora	Patients with NAFLD	[109]
**Probiotic**	Probiotic yogurt	Improving hepatic enzymes, serum TC, and low-density lipoprotein cholesterol levels	72 patients with NAFLD	[121]
		Improvements in total cholesterol and LDL-C concentrations	60 people with type 2 diabetes and low-density lipoprotein cholesterol	[122]

NASH: nonalcoholic steatohepatitis; MCD: methionine-choline-deficient; HFD: high-fat diet; LDL: low-density lipoprotein; HDL-C: low density lipoprotein cholesterol; HDL: high-density lipoprotein; HDL-C: high density lipoprotein cholesterol; TG: triglycerides; TC: total cholesterol; IL: interleukin. T2D: type 2 diabetes; AST: aspartate aminotransferase; ALT: alanine aminotransferase; LPS: lipopolysaccharides; TLR: Toll like receptor; HSHF: high sugar and high fat; TNF-α: tumor-necrosis factor alpha.

**Table 2:** Gut microbiota-targeted therapies of NAFLD with prebiotics.

Interventions		Main Effects	Experimental Models	Ref.
**Prebiotic**	Oligofructose (OFS)	Lowering LPS and cytokine levels, and decreasing the hepatic expression of inflammatory and oxidative stress markers	Obese and diabetic mice	[128]
		Decreasing serum ALT, AST and insulin level	Patients with NASH	[127]
	Fructooligosaccharides (FOS)	Restoring normal gastrointestinal microflora and intestinal epithelial barrier function, and decreasing steatohepatitis	MCD diet-induced NASH in mice.	[129]
		Reducing hepatic TG and TC level, modulating hepatic steatosis	*N*-3PUFA (polyunsaturated fatty acid)-depleted diet-fed mice	[130]
	Lactulose	Ameliorating the hepatic inflammation and decreasing serum levels of ALT and AST	HFD-induced NASH in rats	[133]
	Chitin–glucan (CG)	Decreasing weight gain, fat mass development, glucose intolerance, and hepatic TG accumulation	HFD-induced obese mice	[134]
	Isomalto-oligosaccharides (IMOs)	Preventing weight gain, adiposity, and improving insulin resistance.	HFD-induced NAFLD in mice	[135]
	Galacto-oligosaccharides and fructo-oligosaccharides (9:1)	Increasing abundance and proportion of bifidobacteria	Formula-fed infants (FF)	[141]
	Inulin-type fructans( ITF) prebiotics (inulin + oligofructose)	Changing the gut microbiota composition and host metabolism	30 obese women	[142]

**Table 3:** Gut microbiota-targeted therapies of NAFLD with synbiotics.

Interventions		Main Effects	Experimental Models	Ref.
**Synbiotic**	*L. paracasei* B21060 + arabinogalactan + FOS	Lessening NAFLD progression, preserving gut barrier integrity and reducing the severity of liver injury and IR	HFD-induced NAFLD in rats	[146]
	Seven probiotics + OFS	Improving NAFLD and decreasing levels of ALT and AST	52 patients with NAFLD	[150]
	*B. longum* + FOS	Reductions in TNF-a, serum AST levels, serum endotoxins, steatosis, and the NASH activity index	66 patients with NASH	[151]
	Dietary fiber + *L*. *reuteri*	Improving NAFLD and reducing serum levels of most of the inflammatory mediators	50 lean patients with NAFLD	[152]
	Seven probiotics + FOS	Protecting against NAFLD progression and improving steatosis	80 NAFLD patients	[153]

**Table 4:** Gut microbiota-targeted therapies of NAFLD—other approaches.

Interventions		Main Effects	Experimental Models	Ref.
**Antibiotic**	Cidomycin	Lowering serum levels of ALT, AST and TNF-α and alleviating the severity of NASH	Rats with NASH	[158]
	Vancomycin + Neomycin + Metronidazole + Ampicillin	Adjusting gut microecology and alleviating the lesions of NAFLD	HFD-induced NAFLD in rats	[175]
	Rifaximin	Improving NAFLD and reducing endotoxin and IL-10 levels	42 patients with NAFLD	[159]
**Herbal medicine or natural active ingredient**	2,3,5,4′-tetrahydroxy-stilbene-2-*O*-β-d-glucoside (TSG)	Reversing NAFLD and reducing FFA accumulation, and increasing the protein expression of ZO-1 and occludin	HFD-induced NAFLD in rats	[169]
	Resveratrol	Reducing blood glucose and lipid levels, and lowering both body and visceral adipose weights	HFD-fed mice	[171]
	Qushi Huayu Fang	Reducing body weight, TG and free fatty acids, alleviating hepatic steatosis	HFD-induced NAFLD in rats	[172]
		Enhancing the hepatic anti-oxidative mechanism, decreasing hepatic lipid synthesis, and promoting the regulatory T cell inducing microbiota in the gut	HFD-induced NAFLD in rats	[173]
	Daesiho-tang (DSHT)	Ameliorating body weight gain, body fat, decreasing TC and TG	HFD-fed obese mice	[174]
	Gegen Qinlian Decoction (GQD)	Alleviating T2D, increasing the amounts of beneficial bacteria	187 patients with type 2 diabetes (T2D)	[176]

## Appendix A

Search strategy

- The main source of material was pubmed, and the search keywords used were as follows: “gut microbiota”, “gut flora”, “nonalcoholic fatty liver disease(NAFLD)”, “nonalcoholic steatohepatitis(NASH)”, “steatosis”,“probiotic”, “prebiotic”, “antibiotic”, “herbal medicince”;
- Selected papers have no language restrictions;
- Most of the papers selected were published during the past 10 years;
- References of some identified papers were further searched for related papers to cover this topic as completely as possible.
